# Case-case analysis of enteric diseases with routine surveillance data: Potential use and example results

**DOI:** 10.1186/1742-5573-5-6

**Published:** 2008-10-31

**Authors:** Nick Wilson, Michael Baker, Richard Edwards, Greg Simmons

**Affiliations:** 1 Departament of Public Health, University of Otago, Wellington, New Zealand; 2 Auckland Regional Public Health Service, Auckland, New Zealand

## Abstract

**Background:**

Case-control studies and outbreak investigations are the major epidemiological tools for providing detailed information on enteric disease sources and risk factors, but these investigations can be constrained by cost and logistics.

**Methods:**

We explored the advantages and disadvantages of comparing risk factors for enteric diseases using the case-case method. The main issues are illustrated with an analysis of routine notification data on enteric diseases for 2006 collected by New Zealand's national surveillance system.

**Results:**

Our analyses of aggregated New Zealand surveillance data found that the associations (crude odds ratios) for risk factors of enteric disease were fairly consistent with findings from local case-control studies and outbreak investigations, adding support for the use of the case-case analytical approach. Despite various inherent limitations, such an approach has the potential to contribute to the monitoring of risk factor trends for enteric diseases. Nevertheless, using the case-case method for analysis of routine surveillance data may need to be accompanied by: (i) reduction of potential selection and information biases by improving the quality of the surveillance data; and (ii) reduction of confounding by conducting more sophisticated analyses based on individual-level data.

**Conclusion:**

Case-case analyses of enteric diseases using routine surveillance data might be a useful low-cost means to study trends in enteric disease sources and inform control measures. If used, it should probably supplement rather than replace outbreak investigations and case-control studies. Furthermore, it could be enhanced by utilising high quality individual-level data provided by nationally-representative sentinel sites for enteric disease surveillance.

## Background

In most developed countries, information on enteric disease epidemiology comes from a range of routine sources, outbreak investigations and case-control studies conducted outside of the context of an outbreak. The most accessible information comes from routine compilations of pathogen-specific surveillance data. This source usually provides important information on demographic, temporal and geographic trends and can be used both to identify potential outbreaks and to monitor trends that may suggest potential exposure sources. Maintaining and analysing these data are a routine part of infectious disease surveillance. Outbreak investigations are a necessary extension of public health surveillance and are particularly critical where the source of an outbreak is not well established. However, risk factors and sources of disease identified in the context of an outbreak may not always reflect the major transmission pathways for the disease agent in the general population. Case-control studies aiming to investigate risk factors and sources of disease outside of outbreaks may be the best way to characterise transmission pathways for the disease within populations and for sporadic (non-outbreak) cases. However, such studies are expensive, and go beyond the capacity of many public health agencies.

For example, in the country we are most familiar with (New Zealand), case-control studies have proved useful in identifying risk factors for sporadic enteric infections including campylobacteriosis [1-5], giardiasis [6-9], salmonellosis [10,11], and yersiniosis [12]. However, these case-control studies have been demanding on limited health worker time and public health resources. In these studies recall bias has also been a serious concern and this may be an increasing problem due to greater media publicity around risk factors for common enteric diseases.

A potentially less expensive analytic approach, that may be less susceptible to recall bias, is the case-case method. This is a variant of the case-control design that was first described in the 1980s when applied to cancer epidemiology [13]. In infectious disease epidemiology, the case-case method has usually involved comparisons between cases infected with a different strain (or strains) of the same infectious disease agent and selected from a similar surveillance system [14]. It has been described as a useful tool for communicable disease epidemiology [14] and has been utilised for studying enteric disease outbreaks (eg, salmonellosis [15] and campylobacteriosis [16,17]) and health outcomes from infection with enteric diseases [18]. Here we consider the potential advantages and disadvantages of using the case-case method to identify risk factors for enteric diseases using nationally collected routine surveillance data.

## Methods

We explored the advantages and disadvantages of the case-case method for studying risk factors for enteric diseases using routine surveillance data, through comparisons with other studies (mainly case-control studies). Domains that were considered on the basis of our understanding of the enteric disease epidemiological literature were: (i) selection bias among cases; (ii) selection bias among controls (or comparison cases in this instance); (iii) information and recall bias; (iv) confounding; and (v) lack of detail of exposures.

A worked example of case-case analyses for risk factor comparisons used the routine national notification data on enteric diseases for New Zealand in 2006. These data are collected by the public health services of District Health Boards and published by the Institute of Environmental Science and Research Ltd (ESR), a national disease surveillance and reference laboratory organisation [19]. Comparisons using aggregated national level data were made to assess the associations between nine potential risk factors for which data are routinely collected and six different enteric diseases. Individual level analyses were not possible as we did not have access to the individual level data. Campylobacteriosis was used as the "reference group" (comparison case group) for each case-case analysis since this disease is relatively well studied in New Zealand, has fairly well-established risk factors [2,3,20] and is the most frequently notified disease (so adequate numbers were available for all the analyses) [21]. Crude odds ratios (OR) and 95% confidence intervals were calculated using OpenEpi [22].

Notification data in New Zealand come from medical practitioners who are legally required to report "notifiable diseases" to their local Medical Officer of Health. Some laboratories have also provided this information to health authorities on a voluntary basis (though notification also became a legal requirement for all laboratories in December 2007 under the Health Amendment Act 2006). Standardised forms used for enteric disease notifications include risk factor questions and these are usually completed by public health staff interviewing cases over the telephone. However, due to resource constraints, this risk factor collection is far from complete (see Table 1).

**Table 1:** Reported risk factor exposure and crude odds ratios for different notifiable enteric diseases (using published aggregate national data for 2006 [19])

	Reported exposure to risk factor (RF)*					Crude odds ratio (OR)**	
	**Yes**	**No**	**Unknown**	**% Reported the RF**	**% Unknown**	**OR**	**95% CI**

***Overseas travel***							
Shigellosis	49	32	21	60.5	20.6	**23.9**	**15.1 – 37.8**
Giardiasis	157	434	623	26.6	51.3	**5.6**	**4.5 – 7.0**
Salmonellosis	184	770	381	19.3	28.5	**3.7**	**3.1 – 4.5**
Cryptosporidiosis	43	480	213	8.2	28.9	1.4	1.0 – 1.9
Yersiniosis	19	266	202	6.7	41.5	1.1	0.69 – 1.8
*Campylobacteriosis*	*304*	*4734*	*10,835*	*6.0*	*68.3*	*1.0 (ref)*	

***Food consumption from a food premise***							
Shigellosis	23	24	55	48.9	53.9	0.87	0.49 – 1.5
Salmonellosis	310	364	661	46.0	49.5	**0.77**	**0.66 – 0.91**
Yersiniosis	72	136	279	34.6	57.3	**0.48**	**0.36 – 0.64**
Giardiasis	100	270	844	27.0	69.5	**0.34**	**0.27 – 0.43**
Cryptosporidiosis	79	225	432	26.0	58.7	**0.32**	**0.24 – 0.42**
*Campylobacteriosis*	*2096*	*1903*	*11,874*	*52.4*	*74.8*	*1.0 (ref)*	

***Consumption of untreated drinking water***							
Giardiasis	168	293	753	36.4	62.0	**2.9**	**2.4 – 3.6**
Cryptosporidiosis	152	286	298	34.7	40.5	**2.7**	**2.2 – 3.3**
Salmonellosis	149	588	598	20.2	44.8	**1.3**	**1.1 – 1.6**
Yersiniosis	47	187	253	20.1	52.0	1.3	0.92 – 1.8
Shigellosis	6	39	57	13.3	55.9	0.78	0.33 – 1.9
*Campylobacteriosis*	*679*	*3451*	*11,743*	*16.4*	*74.0*	*1.0 (ref)*	

***Contact with recreational water***							
Cryptosporidiosis	143	337	256	29.8	34.8	**3.5**	**2.8 – 4.4**
Giardiasis	138	359	717	27.8	59.1	**3.2**	**2.6 – 4.0**
Shigellosis	10	35	57	22.2	55.9	**2.4**	**1.2 – 4.8**
Salmonellosis	134	667	534	16.7	40.0	**1.7**	**1.4 – 2.1**
Yersiniosis	29	222	236	11.6	48.5	1.1	0.73 – 1.6
*Campylobacteriosis*	*474*	*3948*	*11,451*	*10.7*	*72.1*	*1.0 (ref)*	

***Contact with farm animals***							
Cryptosporidiosis	284	250	202	53.2	27.4	**2.8**	**2.4 – 3.4**
Yersiniosis	72	136	279	34.6	57.3	1.3	0.99 – 1.8
Giardiasis	139	365	710	27.6	58.5	0.95	0.77 – 1.2
Salmonellosis	217	643	475	25.2	35.6	**0.84**	**0.71 – 0.99**
Shigellosis	2	43	57	4.4	55.9	**0.12**	**0.03 – 0.48**
*Campylobacteriosis*	*1289*	*3215*	*11,369*	*28.6*	*71.6*	*1.0 (ref)*	

***Contact with sick animals***							
Cryptosporidiosis	107	333	296	24.3	40.2	**7.1**	**5.4 – 9.2**
Giardiasis	24	412	778	5.5	64.1	1.3	0.82 – 2.0
Salmonellosis	37	747	551	4.7	41.3	1.1	0.76 – 1.6
Yersiniosis	11	230	246	4.6	50.5	1.0	0.56 – 2.0
Shigellosis	0	45	57	0.0	55.9	0.0	-
*Campylobacteriosis*	*177*	*3884*	*11,812*	*4.4*	*74.4*	*1.0 (ref)*	

***Contact with symptomatic people***							
Giardiasis	146	327	741	30.9	61.0	**3.9**	**3.2 – 4.9**
Cryptosporidiosis	118	365	253	24.4	34.4	**2.9**	**2.3 – 3.6**
Shigellosis	11	46	45	19.3	44.1	**2.1**	**1.1 – 4.1**
Salmonellosis	107	705	523	13.2	39.2	**1.3**	**1.1 – 1.7**
Yersiniosis	24	222	241	9.8	49.5	0.95	0.61 – 1.5
*Campylobacteriosis*	*442*	*3901*	*11,530*	*10.2*	*72.6*	*1.0 (ref)*	

***Contact with confirmed cases (of the same specific disease)***							
Giardiasis	108	414	692	20.7	57.0	**7.3**	**5.7 – 9.5**
Shigellosis	8	49	45	14.0	44.1	**4.6**	**2.1 – 9.8**
Cryptosporidiosis	27	417	292	6.1	39.7	**1.8**	**1.2 – 2.8**
Salmonellosis	35	739	561	4.5	42.0	1.3	0.92 – 1.9
Yersiniosis	1	201	285	0.5	58.5	0.14	0.02 – 1.0
*Campylobacteriosis*	*185*	*5210*	*10,478*	*3.4*	*66.0*	*1.0 (ref)*	

***Contact with human faecal matter***							
Giardiasis	151	324	739	31.8	60.9	**3.6**	**2.9 – 4.4**
Cryptosporidiosis	151	343	242	30.6	32.9	**3.4**	**2.7 – 4.2**
Yersiniosis	49	202	236	19.5	48.5	**1.9**	**1.3 – 2.6**
Salmonellosis	117	701	517	14.3	38.7	1.3	1.0 – 1.6
Shigellosis	3	49	50	5.8	49.0	0.47	0.15 – 1.5
*Campylobacteriosis*	*504*	*3850*	*11,519*	*11.6*	*72.6*	*1.0 (ref)*	

Notes:

* For within the incubation period of each particular disease.

** Crude odds ratios not adjusted for sex, age, ethnicity or any other covariates.

## Results

### Specific results from the worked example

The results for the nine risk factors for which data are routinely collected are detailed in Table 1. Given the likely methodological limitations of the data and this analysis (see below) we only present these specific results for illustrative purposes and they should not be used on their own for any decision-making by disease control agencies.

Significantly elevated crude odds ratios were apparent for overseas travel and shigellosis (OR = 24), giardiasis (OR = 5.6) and salmonellosis (OR = 3.7). Elevated crude OR were also evident for consumption of untreated drinking water for giardiasis (OR = 2.9), cryptosporidiosis (OR = 2.7) and salmonellosis (OR = 1.3). For the latter three diseases, this pattern was also evident for contact with recreational water (as it was for shigellosis as well).

Contact with farm animals and contact with sick animals were apparent risk factors for cryptosporidiosis (OR = 2.8 and OR = 7.1 respectively). For contact with symptomatic people all diseases except yersiniosis had elevated OR. This pattern also held for contact with confirmed cases (except it was no longer significant for salmonellosis). Contact with human faecal matter had elevated OR for all diseases except salmonellosis and shigellosis.

There were some diseases where the OR were significantly lower than for the reference disease of campylobacteriosis. That pattern was seen for all the diseases, except shigellosis, for consumption of food from a food premise; and for both salmonellosis and shigellosis for contact with farm animals. These findings may suggest that these risk factors are significantly more important for campylobacteriosis than for these other diseases in the New Zealand context.

### Limitations of case-case analyses using surveillance data

Our comparative analyses of enteric disease risk factors using the case-case method and routine surveillance data identified some important limitations with this approach. These limitations are illustrated with examples drawn from New Zealand's infectious diseases surveillance systems:

#### 1) Selection bias among cases

A possible selection bias is that risk factor exposures among the study cases may be atypical because the cases are not representative of all cases in the population (indeed probably only a small proportion of all cases [23]). For example, the cases may tend to be more severe with more serious symptoms that increase the likelihood of them seeking medical attention. In New Zealand few enteric disease cases are likely to be notified on suspicion to the local health authorities (the District Health Boards in NZ), and most will therefore be laboratory-confirmed. For laboratory-confirmed cases of enteric disease, there are likely to be large selection biases associated with the type of people who seek medical advice when they have gastrointestinal symptoms, from whom a stool specimen is requested by a doctor and then who actually provide a stool specimen to a laboratory to confirm the cause [23-25]. Furthermore, under the current 'passive' system of disease notification in New Zealand, one study reported that 23% of laboratory-confirmed cases of enteric disease were not notified (range: 12% for shigellosis 24% for campylobacteriosis [26]), so further unknown selection biases may operate for laboratory-confirmed cases which are notified. However, with recent legislation requiring mandatory laboratory notification nation-wide, this situation should improve.

Similar selection bias may also occur in case-control studies which use notified or laboratory-confirmed cases. Arguably, there is greater potential for selection bias with case-control studies where cases are derived from notifications but where controls are recruited from a difference source (commonly from the general population via telephone in New Zealand).

#### 2) Selection bias among controls or comparison cases

A specific limitation of case-case analysis is that the estimated strength of the exposure-disease association is strongly influenced by the level of exposure reported by the comparison cases. If the exposure was a protective factor for the comparison cases (those with the reference disease), then this analysis would tend to over-estimate the strength of association. By contrast, if the exposure is also a risk factor for the reference disease, then this type of case-case analysis will under-estimate the strength of the association. Changes in the pattern of exposure among the comparison cases could result in spurious changes to the estimated exposure-disease association, and this possible explanation should be considered where associations change over time. However, given that the exposures reported for these diseases are all well-established risk factors for most enteric diseases, results from this risk factor comparative analysis will tend to be conservative (ie, biased towards not showing an association). Examples of this are the results for "overseas travel" and for "food consumption from a food premise" (Table 1) where these risk factors are already known for campylobacteriosis in the New Zealand setting [2,20].

#### 3) Information/recall biases

Information biases may occur in a case-case analysis due to biased investigator data collection or respondent recall of exposure. The systems for collecting risk factor information from cases of notifiable enteric diseases are often heterogeneous, particularly in countries where health systems vary at district or regional levels. For example, in New Zealand information collection can involve interviews by communicable disease clerks, health protection officers or even self-completion of printed questionnaires by cases. Both case investigators and cases are usually aware of the specific enteric disease diagnosis when risk factor data are being collected. Information bias may therefore occur as some of these risk factors may be well known by case investigators and cases themselves (eg, campylobacteriosis and food; giardiasis and untreated water). This situation could result in improved or even false recall of exposure to specific well-known risk factors or to exposures which are widely viewed as risk factors for enteric infections, increasing the observed association with the disease. However, as for case-case studies, case-control studies are also likely to be affected by such recall biases. Indeed, recall bias is likely to be a larger problem for case-control analyses involving community controls, as such controls will not have had a gastro-intestinal infection to stimulate recall of known or suspected causes of these diseases.

#### 4) Confounding

There are numerous potential confounders in any case-case analysis using aggregated routinely collected enteric disease surveillance data (eg, age, gender, socioeconomic status, ethnicity, rurality, month of infection and variable length of incubation period for the different diseases). These effects may be reduced through using comparison cases with other laboratory-confirmed enteric infections, though this is certainly not guaranteed. Appropriate adjustment for these potential confounders using individual data may well be worthwhile once improvements in surveillance data quality are made (eg, the proportion of "unknown" responses to each exposure in Table 1 is reduced). Indeed, in many jurisdictions it is possible that the address data of cases could be used to obtain a measure of socioeconomic position for each case (eg, in New Zealand using "NZDep", a small area measure of social deprivation [27]).

#### 5) Lack of detail of exposures

The scope of specific exposures recorded in routine surveillance data is always going to be restricted and in the New Zealand example it was limited to just nine. Furthermore, some of these risk factors were quite broad in nature (eg, *Food consumption from a food premise)*. Consequently, the findings do not provide a particularly strong basis for taking specific public health action to reduce exposures to these sources. This limitation could be reduced by improved data collection with greater detail, specificity and completeness of the exposure data. This expansion could extend to a "shotgun" type questionnaire to provide a tool for rapid hypothesis generation for use in outbreak situations (eg, as used by the Oregon State Department of Human Services: ). The obvious trade-off would be the additional time and resources required to obtain detailed exposure history information. As noted later in the *Discussion Section*, this extension could be facilitated through the use of sentinel data collection sites.

### Strengths of case-case analyses using surveillance data

As described above, many of the limitations of case-case analyses for enteric diseases are common to case-control studies. Both share the limitations with what participants can observe and report, the long exposure windows for some pathogens, and the uncertain effects of population immunity for some of these enteric diseases [28,29]. However, the recall bias problem is probably greater with case-control studies so that is a relative strength of the case-case approach.

Another strength of the case-case approach over the case-control one is that by using routinely collected data it is potentially much less expensive. This cost barrier may partly explain why case-control studies are done infrequently. For example, in New Zealand there has never been a reported case-control study investigating sporadic (non-outbreak) cryptosporidiosis and shigellosis. Also, by being easier to undertake, these case-case analyses may be more readily made a routine part of ongoing national surveillance activities. Indeed, community controls for case-control studies are becoming very difficult and expensive to obtain due to the low participation rate from telephone recruitment methods (eg, only 21.4% in a recent New Zealand study [30]).

A further relative advantage of the case-case approach could be timeliness as the analyses can use already collected case data. This requirement is particularly important in some rapidly evolving outbreaks, such as may occur where a widely distributed food is contaminated with a serious disease causing agent such as verotoxigenic *E. coli *[31]. Control of such outbreaks depends on swiftly generating and investigating hypotheses about the source of the infection. So rather than expending weeks collecting data from controls, the historical case data from the surveillance system could be analysed quickly (with the new case data) for an initial case-case investigation to see if any of the major risk factors are involved.

## Discussion

### Interpretation of the example results

For the worked example using New Zealand surveillance data it is apparent that the associations are fairly consistent with other published New Zealand evidence about risk factors for specific enteric diseases eg:

• Overseas travel and shigellosis based on an outbreak investigation [32] and also giardiasis based on two case-control studies [7,9].

• Drinking water and giardiasis based on case-control studies [6-9] and also plausible for cryptosporidiosis given the results of a cross-sectional survey [33].

• Contact with farm animals and cryptosporidiosis based on an outbreak investigation [34]. Of note however, is the possibility that outbreaks sometimes reflect atypical exposure pathways and so can produce different findings from analytic epidemiological studies involving sporadic cases.

• Contact with confirmed cases and giardiasis has not been specifically assessed in previous New Zealand studies but the risk associated with human waste exposure is well documented for this country based on case-control studies [7,9,35] which suggests that some forms of human-to-human transmission may be important in this setting.

The finding that there were some diseases with an OR that was statistically significantly lower than for the reference disease of campylobacteriosis was also not surprising. That is because there is good evidence from many studies that contaminated food (especially fresh poultry) is an important risk factor for campylobacteriosis in New Zealand [20,36].

The findings therefore suggest that case-case analysis using routine data may provide supplementary information on risk factors for enteric diseases. Repeated over time, this approach could potentially provide further information on progress with disease prevention interventions (eg, as rural water supply quality in New Zealand continues to improve, the associated elevated risks for consuming untreated water should decline over time). But interpreting apparent trends for an enteric disease would need a lot of additional contextual information, including changes in exposure experienced by the comparison cases with the reference disease(s).

It is also conceivable that these case-case analyses could identify newly emerging risk factors if repeated periodically (six-monthly or annually) and act as a trigger for more in-depth investigations. Nevertheless, we would argue against any of these routine uses of such case-case analyses with current New Zealand data until further work is done to assess the importance of the biases detailed in the *Results Section*.

### Implications for improving surveillance data

Given the considerations detailed in the preceding *Results Section*, we recommend that countries wishing to undertake supplementary case-case analyses with routine enteric disease surveillance data ensure that they have high quality data collection processes. For New Zealand, this would mean reducing the large proportions of "unknowns" as listed in Table 1 and improving the availability of data on potential confounding factors. The most efficient way to do this may be to establish a number of appropriately resourced specialised sentinel surveillance sites that collect more comprehensive and complete information.

Sentinel surveillance is "surveillance based on selected population samples chosen to represent the relevant experiences of particular groups" [37]. This approach is used where the health event is very common and it would be impractical to record every case, or where more intense effort is used to collect additional data on a sub-sample of cases. In New Zealand, sentinel surveillance is well established for monitoring seasonal influenza where general practitioners provide data on patients consulting them for influenza-like illness [38]. General practice sentinel surveillance has also been successfully used on a trial basis to collect data on acute gastroenteritis presenting to general practitioners in New Zealand [39]. Elsewhere, ongoing sentinel surveillance of gastroenteritis does not appear to be widely used. One of the few reported examples is the French sentinel surveillance system which includes acute diarrhoea as one of the conditions under surveillance [40].

We are not aware of New Zealand examples of sentinel surveillance where additional exposure or risk factor information is collected on a sample of cases on an ongoing basis. However, there are several diseases where considerable risk factor/exposure data are collected on all cases, with HIV/AIDS being probably the best example [41]. Similarly, we are not aware of international examples of ongoing sentinel surveillance of gastroenteritis that collects risk factor or exposure information beyond basic demographic and possibly travel history information. More intensive surveillance of this type has, however, been successfully used as part of specific research studies on intestinal infectious diseases in the United Kingdom [23,24] and The Netherlands [25].

Developing sentinel surveillance system for gastrointestinal illness could reduce most of the limitations of case-case analyses described above (the one exception being selection bias among comparison cases). Sentinel surveillance could reduce selection bias among cases by putting greater effort into obtaining faecal specimens from patients presenting with gastroenteritis. Information/recall biases could be reduced by shortening the time delay before interviewing cases (potentially interviewing cases while waiting for laboratory test results). Confounding could be reduced by collecting more complete data from cases allowing for adjusted analyses of individual level data. Lack of detail of exposures could be reduced through the use of highly detailed questionnaires covering important disease sources (the "shotgun" type questionnaires referred to above).

Some of the extra cost of running these sites could be offset by a reduced need for collecting routine risk factor data in the non-sentinel areas (eg, on more common enteric diseases such campylobacteriosis). If sentinel sites were appropriately resourced then they could also run case-control studies, case-case studies (between strains) and case-crossover studies that would allow direct comparisons with the cruder case-case method used here. For case-control studies the controls could possibly be selected from the general practices involved in notifying the cases (given the difficulty in recruiting community controls in many developed countries). This approach could provide an established population of controls to facilitation rapid outbreak investigation ie a "control bank" [42,43].

## Conclusion

Despite various inherent limitations, case-case analyses using routine surveillance data have the potential to supplement other studies to assist in monitoring risk factor trends for enteric diseases. They may also help with monitoring the impact of interventions and could potentially provide a base for rapid outbreak investigations. Nevertheless, adopting this approach may need to be accompanied by moves to: (i) reduce potential selection and information biases by improving the quality of the surveillance data; and (ii) reduce confounding by undertaking more sophisticated analyses of individual data. These improvements could be facilitated by establishing high quality sentinel sites for enteric disease surveillance. For the particular New Zealand data studied, it was apparent that the case-case analyses of enteric diseases data produced information that was consistent with findings from local case-control studies and outbreak investigations. That observation adds support for the use of this type of case-case analysis for investigating sources and studying the impact of control measures for enteric diseases.

## Competing interests

The lead author (NW) has previously undertaken contract work in 2005 for the government agency involved in foodborne disease control (the NZ Food Safety Authority). The other authors declare that they have no competing interests.

## Authors' contributions

NW was responsible for data collation and analysis. All authors were involved in interpreting the results and drafting various versions of the article. All authors read and approved the final manuscript.
